# CRISPR/Cas9-mediated knockout of APOC3 stabilizes plasma lipids and inhibits atherosclerosis in rabbits

**DOI:** 10.1186/s12944-021-01605-7

**Published:** 2021-12-18

**Authors:** Yiwen Zha, Yaoyao Lu, Ting Zhang, Kunning Yan, Wenwen Zhuang, Jingyan Liang, Yong Cheng, Yingge Wang

**Affiliations:** 1 Medical College, Yangzhou University, Yangzhou, 225001 Jiangsu China; 2 College of Veterinary Medicine, Yangzhou University, Yangzhou, 225009 Jiangsu China; 3 Institute of Translational Medicine, Medical College, Yangzhou University, Yangzhou, 225001 Jiangsu China; 4 Jiangsu Key Laboratory of Integrated Traditional Chinese and Western Medicine for Prevention and Treatment of Senile Diseases, Yangzhou University, Yangzhou, 225001 China; 5 Jiangsu Co-Innovation Center for Prevention and Control of Important Animal Infectious Disease and Zoonoses, Yangzhou, 225001 China; 6 Affiliated Hospital of Yangzhou University, Yangzhou, 225001 Jiangsu China

**Keywords:** Apolipoprotein, Triglycerides, Atherosclerosis, CRISPR/Cas9, Rabbit

## Abstract

**Background:**

High levels of apolipoprotein C3 (APOC3) can lead to hypertriglyceridemia, which increases the risk of cardiovascular disease. We aim to create APOC3-knockout (KO) rabbits and explore the effects of APOC3 deletion on the occurrence and development of atherosclerosis.

**Methods:**

An sgRNA anchored to exon 2 of APOC3 was designed to edit embryo genomes using the CRISPR/Cas9 system. The founder rabbits were sequenced, and their lipid profile, inflammatory cytokines, and atherosclerotic plaques were analyzed.

**Results:**

When given a normal chow (NC) diet, all APOC3-KO rabbits had 50% lower triglyceride (TG) levels than those of the matched age control group. Additionally, their plasma lipoprotein lipase increased. When fed a high-fat diet, APOC3 deficiency was observed to be more conducive to the maintenance of plasma TG, total cholesterol, and low-density lipoprotein cholesterol levels, and the inhibition of the inflammatory response and the protection against atherosclerosis in rabbits.

**Conclusion:**

APOC3 deficiency can delay the formation of atherosclerosis-induced HFD in rabbits, indicating this is a novel therapeutic target to treat atherosclerosis.

**Supplementary Information:**

The online version contains supplementary material available at 10.1186/s12944-021-01605-7.

## Introduction

APOC3 is a key regulator of plasma triglycerides (TG) and shows significant correlation with plasma very low-density lipoprotein (VLDL) levels [1, 2]. Hypertriglyceridemia represents an independent risk factor for cardio- and cerebrovascular diseases [3, 4]. Although in vivo studies on APOC3 have been mainly based on mouse models, immense differences in lipid metabolism have been shown to exist between mice and humans. Carriers with APOC3 mutations had a 40% lower risk of coronary heart disease [5], but no protective effect of APOC3 deficiency on atherogenesis was observed in knockout (KO) mice [6]. Mice secrete VLDL containing either apolipoprotein B100 (Apob100) or apolipoprotein B48 (Apob48), while human and rabbit VLDL only contains APOB100. As opposed to mice, in which high-density lipoprotein (HDL) is the main plasma lipoprotein, rabbits and humans have LDL-rich lipoprotein profiles [7]. All animal models of human disease have strengths and limitations, and domestic rabbits (*Oryctolagus cuniculus*) continue to play significant roles in the study of lipid metabolism and histopathology [8].

CRISPR/Cas9 is machinery for editing genomes that forms a natural part of the bacterial defense mechanism and has been developed as a tool for genetic manipulation in mice [9], rabbits [10, 11], dogs [12], hamsters [13], etc. In comparison with earlier genome-editing technologies, e.g., zinc finger nucleases (ZFNs) and transcription activator-like effector nucleases [14], CRISPR/Cas9 has superior targeting efficiency, is less expensive, and is easier to design and implement [15]. In addition, CRISPR-mediated genome editing is emerging as a therapeutic strategy for combating cardiovascular diseases [16]. We designed our exon 2-anchored CRISPR/Cas9 system to generate APOC3-KO rabbits with biallelic mutations to study the relationship between APOC3 deficiency, abnormal lipid metabolism, and the formation and development of atherosclerosis in this study.

## Material and methods

### Animals

New Zealand White rabbits were sourced from Yangzhou University’s Animal Genetic Engineering Laboratory. The animals were kept in a barrier facility with a 12-h light–dark cycle at 24 °C and 55% humidity, and they were provided ad libitum access to water and fed twice a day with normal chow (NC) diet or high-fat diet (HFD) (TP 2R301, Trophic, Ltd., Nantong, China). Yangzhou University’s Animal Care Committee approved all animal studies in this work.

### Cas9 mRNA and sgRNA preparation

**Fig. 1:**
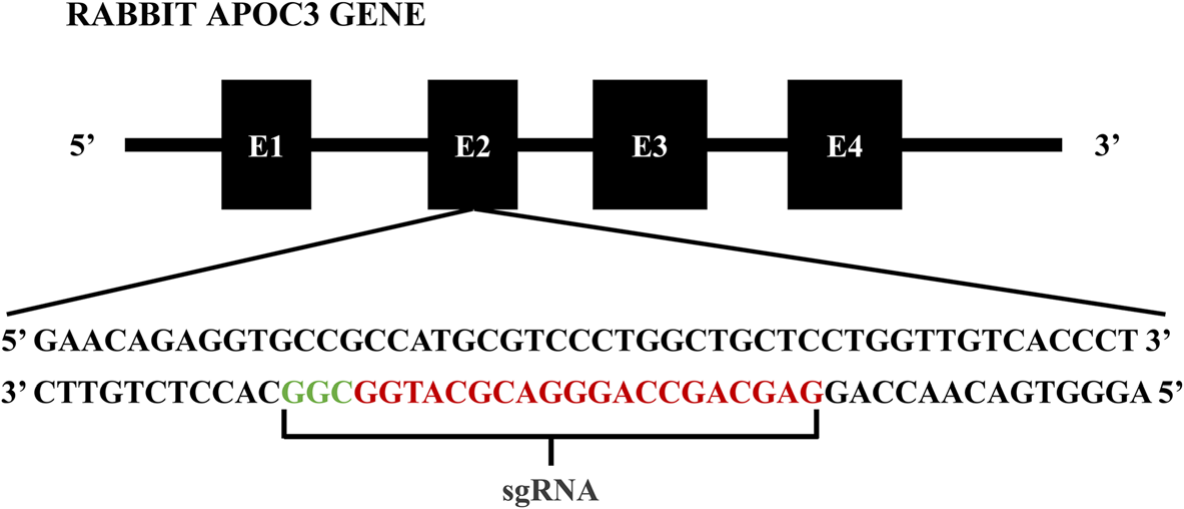
Schematic illustration of CRISPR/Cas9-targeting sites of rabbit APOC3. An sgRNA was designed for anchoring to APOC3 exon 2. The sgRNA-targeting sequence is indicated in red, and the protospacer adjacent motif (PAM) sequence is indicated in green

To design mutant loci of the rabbit APOC3 gene, we obtained sequences from NCBI (http://www.ncbi.nlm.nih.gov/) and designed CRISPR/Cas9 single-guide (sg) RNAs using the tool provided on the website: http://crispr.mit.edu. Then, we chose one sgRNA targeting the loci of rabbit APOC3 by anchoring to exon 2, as shown in Fig. 1.

### Zygote injection and embryo transplantation

Female rabbits older than 6 months and fertile male rabbits were used to obtain embryos. Donor rabbits were superovulated with 60 IU pregnant mare serum gonadotropin, and 72 h later, the donors and recipients were simultaneously administered 10 IU human chorionic gonadotropin intravenously, and the donors were mated with male rabbits. To collect embryos for microinjection, they were aspirated 18–20 h after mating using PBS. Under a Leica inverted light microscope, the embryo cytoplasms were microinjected with a mix containing Cas9 mRNA (33 ng/μl) and sgRNA (40 ng/μl) in solution. The processed embryos were placed in M2 cushion fluid and incubated at 38 °C, 5% CO2 for 30 min, and the oviducts of pseudo-pregnant female rabbits received 10–15 of the resulting zygotes.

### Sequencing analysis of founder rabbits

Tail biopsy specimens were taken for the extraction of genomic DNA using the phenol-chloroform method. PCR amplification of sgRNA target sites utilized the primers F: 5′-GCTCACCCAGCTGAGATCCAT-3′ and R: 5′-CAAAGTGCTTACGGGCAGAGG-3′. After purifying with agarose gel electrophoresis, the PCR products were cloned into a pMDTM 19-T vector (Takara Bio, Inc., Japan), and Lasergene software (DNASTAR, Inc., USA) was employed for the sequencing and analysis of positive clones.

### Extraction and analysis of mRNA in liver and small intestine

TRIzol reagent (Gibco; Thermo Fisher Scientific, Inc.) was used to extract the total liver and small intestine RNA, which was reverse-transcribed with the iScript cDNA Synthesis Kit (Bio-Rad, CA, USA). In qPCR, the Mini Opticon Real-Time PCR detection system (Bio-Rad) was used with GoTaq qPCR Master Mix (Promega, WI, USA) to amplify specific cDNA. The relative mRNA quantity was calculated using the 2 − ΔΔCq method and normalized to GAPDH. We completed triplicate qPCR reactions on three separate samples.

The sequences of the primers were as follows: APOC3: For 5′′-CCTCCCTTCTCAGCGTCATG-3′, Rev. 5′-GTCCCAGAACCCAGAGAACT-3′ and GADPH: For 5′-CATGTTTGTGATGGGCGTGAACAA-3′, Rev. 3′-TAAGCAGTTGGTGGTGCAGGAT-3′.

### Off-target assay

The CRISPR/Cas9 system is used to efficiently alter genes in living cells and organisms allowing any subsequent changes to the phenotype to be studied. However, certain off-target effects of this technology should be considered. The APOC3-KO rabbits were checked for off-target mutations by first predicting potential off-target sites (POTS) in the sgRNAs with the CRISPR Design Tool (http://crispr.mit.edu/), then selecting the top five POTS for each sgRNA, which were PCR amplified with the primers shown in Table 1 and Sanger sequenced.

**Table 1:** Primers for detection of possible off-target sites

Primers	off-targets of GCAGGGCCAGAGCCCAGGTG AGG
off-targets-1	5′ TCGTCGGCAGCGTCACAAAGTGCTTACGGGCAGA 3’ 5′ GTCTCGTGGGCTCGGCTCCACTTTCCTCCCTGCAG 3’
off-targets-2	5′ TCGTCGGCAGCGTCGAGAAGAAGCACCACCCCTC 3’ 5′ GTCTCGTGGGCTCGGACAGGGATCAAGGAAGGACTG 3’
off-targets-3	5′ TCGTCGGCAGCGTCGGAAATGGTGAGTGAGCCCA 3’ 5′ GTCTCGTGGGCTCGGCAGTCACACGGCTTAGTCGT 3’
off-targets-4	5′ TCGTCGGCAGCGTCTCGTGAAGGACTCTCCACCA 3’ 5′ GTCTCGTGGGCTCGGAATCTCACCTTCGACAGCCG 3’
off-targets-5	5′ TCGTCGGCAGCGTCCGACAGGGGTGGGGAAAC 3’ 5′ GTCTCGTGGGCTCGGTTTCCCAGAAACCTGCCCTC 3’

### Phenotypic examinations

#### Analysis of lipids and apolipoproteins

Samples of EDTA plasma were taken after 12 h of fasting. The plasma total cholesterol (TC), triglyceride (TG), LDL-cholesterol (LDL-C), and HDL-cholesterol (HDL-C) levels were measured enzymatically with commercially available kits (Wako Pure Chemical Industries, Osaka, Japan). Western blotting was used to detect plasma APOC3, APOA1, APOE, and PCSK9; the following primary antibodies were used: goat anti-APOC3 (Genecreate, Wuhan, China), sheep anti-apoA1 (Bio-Rad AbD Serotec, Kidlington, UK), goat anti-APOE (Rockland Inc., Limerick, PA, USA), and mouse anti-PCSK9 (Abcam, Cambridge, MA, USA) polyclonal antibodies. Transferrin (Abcam, Cambridge, UK) was included as a loading control. The secondary antibodies were horseradish-peroxidase-conjugated donkey anti-goat IgG (Jackson Immuno Research Laboratories, West Grove, PA, USA), donkey anti-sheep IgG (Chemicon, Temecula, CA, USA), and goat anti-mouse IgG (Sangon Biotech, Shanghai, China).

### Analysis of plasma lipoprotein profiles

Plasma lipoprotein fractions were determined by agarose gel electrophoresis. Plasma (2 μl) samples were run on 1% agarose gel (Helena Laboratories, Saitama, Japan) electrophoresis and the neutral lipids stained with Fat Red 7B.

### Analysis of plasma HL, LPL

The activities of hepaticlipase (HL) and LPL were assayed using commercial kits (Solarbio, Beijing, China).

### Analysis of plasma inflammation

Plasma levels of interleukin (IL)-1β and tumor necrosis factor alpha (TNF-α) were ascertained using anti-rabbit ELISA (IL-1β, Elabscience, Ltd., Wuhan, China; TNF-α, Cusabio, Wuhan, China). Complete blood counts were measured using a BC-2800Vet auto hematology analyzer (Mindray Medical International Ltd., Shenzhen, China).

### Analysis of fat tolerance

The rabbits were fasted for 12 h, weighed, and orally gavaged with olive oil (Aladdin) at 10 ml.

per kg body weight using a 20 ml syringe. Blood was collected before and after 1, 3, 5, and 7 h of.

gavage, and the plasma TG levels at each time point were measured as already described. Oral fat tolerance tests (OFTT) were evaluated in terms of the accumulation and clearance of TG.

### Analysis of atherosclerosis and liver pathological changes

We analyzed the atherosclerotic lesions of the aorta using a previously published protocol [17]. In brief, the rabbits were sacrificed by lethal sodium pentobarbital injection. Their aortic trees were isolated and longitudinally opened and, after 24-h formalin fixation, stained using Sudan IV (Solarbio Life Science). Areas that stained positive were quantified using Image J and expressed as the percentage of the total tissue area.

In subsequent histological analysis, the aortic root samples were paraffin-sectioned, treated with hematoxylin-eosin (HE) and Masson’s trichrome (MT) stains, and incubated with monoclonal antibodies specific for macrophages (Mφ) (clone: RAM11, Dako, CA, US) or the α-smooth muscle actin in smooth muscle cells (clone: HHF35, Dako).

In accordance with the method described in a published report, we sectioned the formalin-fixed hearts into six blocks and embedded these in paraffin [18]. The left coronary artery was dissected to reveal cross-sections (5 μm/slice) and stained with HE. After formalin fixing the fresh liver tissues for 48 h, they were routinely embedded in paraffin, cut into 5-μm serial sections, and processed for HE staining. To observe lipid accumulation, frozen sections of liver were stained with Oil-red O.

### Statistical analysis

The data were expressed as mean ± SD and compared by Student’s t test. All statistical analyses were conducted on GraphPad Prism, and *P* < 0.05 was used as statistical significance threshold.

## Results

### Production of APOC3 KO rabbits and genotype assay

We designed one sgRNA targeting the APOC3 anchored to exon 2 to generate mutant rabbits. Following in vitro transcription from the sgRNAa, the mRNAs and Cas9 mRNAs were co-injected into the rabbit zygotes. As shown in Tables 2, 144 embryos were injected with Cas9 mRNA, 101 of which were transferred to five pseudo-pregnant recipient rabbits (16–20 embryos per recipient). Three recipient rabbits became pregnant, and five pups were born (numbered AC1-AC5), two of which died on the day of birth (AC4 and AC5). All of the rabbits born (five in all) were sequenced. The three surviving founders were used for further phenotypic testing.

**Table 2:** Summary of Apoc3-KO rabbits generation and gene targeting efficiency

Number of microinjection	NO.1	NO.2	NO.3
No. of injected embryos	51	56	37
No. of transferred embryos	43	38	20
No. of recipients	2	2	1
No. of pregnancies	2	1	0
No. of births in total	3	2	0
No. of live pups	2	1	0
No. of mutants	4	1	0
Rate of mutations (%)	100		

**Fig. 2:**
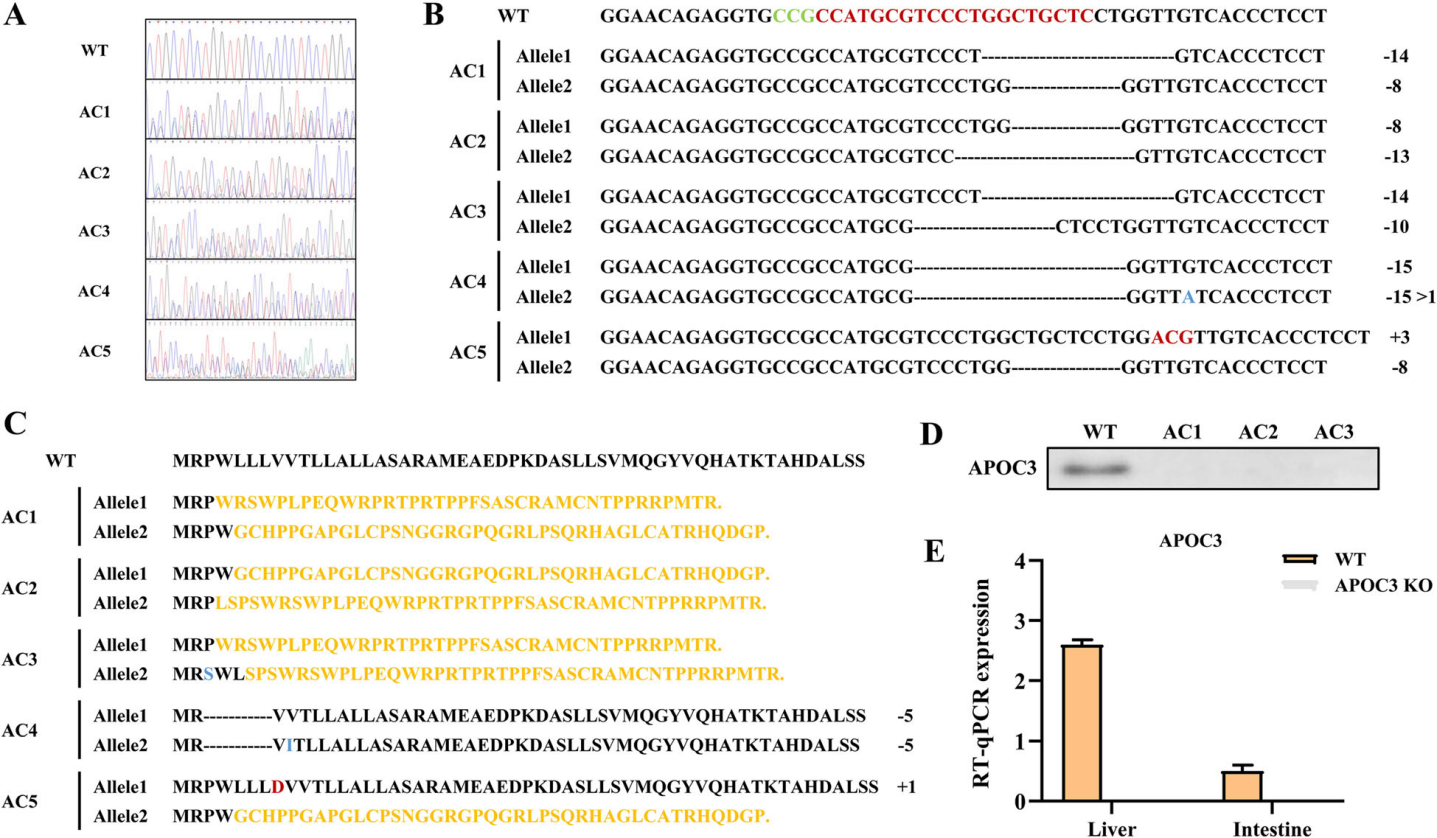
Identification of APOC3 knockout (KO) in rabbits. **a** Sanger sequencing peak map of the rabbits as visualized by Chromas software. **b** Genomic sequence of five mutant rabbits. Deletions are indicated by dotted lines, insertions are indicated in red, and substitutions are indicated in blue. The sizes of the deletions (−), insertions (+), and substitutions (>) are shown in the right column. **c** Theoretical amino acid sequences of five mutant rabbits. Deletions are indicated by dotted lines, insertions are indicated in red, substitutions are indicated in blue, and the frameshift mutations are indicated in orange. The sizes of the deletions (−), insertions (+), and substitutions (>) are shown in the right column. **d** Immunoblot showing presence of the 8.8 kDa APOC3 protein in the plasma. **e** The content of APOC3 in liver and small intestine among WT and mutant rabbits

A pair of primers was designed to amplify the target fragment of APOC3 (F:5′-GCTCACCCAGCTGAGATCCAT-3′, R:5′-CAAAGTGCTTACGGGCAGAGG-3′), which was sequenced to determine whether the gene was mutated; then the PCR products were sequenced by TA-clone to further determine the mutation type and efficiency. The PCR results in Fig. 2A revealed overlapping peaks for all five rabbits, suggesting that there may have been mutations, such as deletions, insertions, or frameshifts. The PCR products were ligated into the pMDTM 19-T vector, and the genomic DNA sequences are shown in Fig. 2B. The theoretical amino acid sequences of the APOC3 mutant alleles are provided in Fig. 2C. AC1♂, AC2♀, and AC3♀ had frameshift mutations that ultimately led to the termination of translation. The amino acid profiles of AC1(1) and AC3(1) were identical, as were the amino acids of AC1(2) and AC2(1).

Western blotting analysis showed that APOC3 was undetectable in the serum of the APOC3-KO rabbits (Fig. 2D). Furthermore, on measuring the RNA levels by RT-qPCR, APOC3 was still detectable in the WT rabbit liver and small intestine, but expression was not detected in the founder rabbits (Fig. 2E). Finally, a total of five POTS were successfully PCR-amplified and Sanger sequenced, which did not reveal any overlapping peaks around the POTS. We concluded that genetic KO of APOC3 was achieved in the rabbit model by CRISPR/Cas9 gene editing technology.

### Analysis of lipids, apolipoproteins, and lipoproteins

Evidence shows that APOC3 can effectively increase the level of circulating TG [19–21]. When the APOC3-KO rabbits were 2 months old, fasting blood samples were collected for lipid concentration detection. As predicted, the KO rabbits showed significantly lower plasma TG levels and higher plasma HDL-C levels than control rabbits of the same age under an NC diet (Table 3). However, there were no significant differences in plasma TC or LDL-C levels between the two groups.

**Table 3:** Plasma levels of TC, TG, LDL-C, and HDL-C of the rabbits

Diet	Group	TC (mg/dl)	TG (mg/dl)	LDL-C (mg/dl)	HDL-C (mg/dl)
NC	WT (*n* = 5)	48.0 ± 8.1	59.9 ± 13.2	23.7 ± 2.9	28.7 ± 4.4
	AC1♂	44.6 ± 7.5	23.8 ± 11.8	18.4 ± 2.7	31.1 ± 5.1
	AC2♀	30.8 ± 6.9	36.3 ± 8.7	18.6 ± 3.1	21.5 ± 6.3
	AC3♀	54.3 ± 8.8	30.5 ± 6.5	20.3 ± 2.6	33.7 ± 3.8
HF (6 W)	WT (*n* = 5)	786.6 ± 48.5	877.4 ± 69.8	329.9 ± 23.3	26.4 ± 9.6
	AC1♂	288.5	256.0	125.3	43.8
	AC2♀	318.8	285.7	159.3	45.1
	AC3♀	355.3	298.5	201.2	39.2
HF (12 W)	WT (*n* = 5)	805.3 ± 39.7	890.7 ± 46.9	403.1 ± 49.3	25.1 ± 3.8
	AC1♂	316.6	224.5	221.4	37.6
	AC2♀	436.1	297.1	226.0	40.3
	AC3♀	399.0	308.3	239.5	38.5

**Fig. 3:**
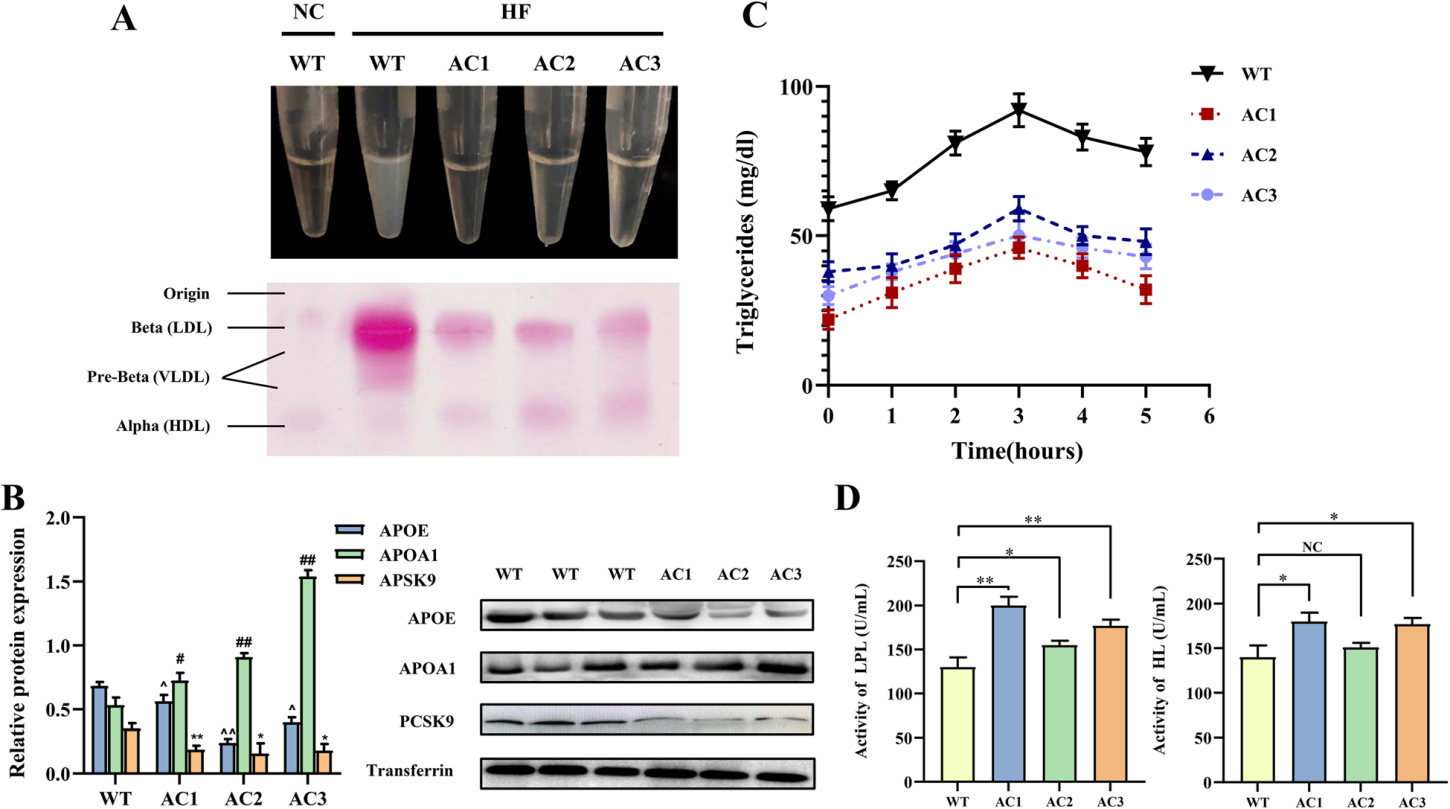
Analysis of plasma lipids, apolipoproteins, and lipoproteins in APOC3-KO rabbits. **a** Agarose gel electrophoresis of plasma lipoproteins; 2 μl of plasma was loaded into each well, fractionated on 1% agarose gel, and stained with fat red 7B for neutral lipids. The corresponding lipoprotein migration positions are marked on the left. **b** Analysis of plasma apolipoproteins by western blotting. Rabbit plasma samples (8 μL) were electrophoresed on SDS-PAGE using 10% gels, transferred to a cellulose membrane, and probed with appropriate antibodies. ^, ^#^ and *: The content of plasma APOE, APOA1 and PCSK9 in AC1-AC3 compared with WT **c** Postprandial plasma TG levels at 0, 1, 2, 3, 4, and 5 h in rabbits following olive oil gavage, as measured by OFTT. OFTT was repeated 3 times, and the average results were selected for statistical analysis. **d** Plasma LPL and HL activity after 12 weeks of FHD. Blood samples were collected three times for enzyme activity analysis. Values are expressed as means ± SD., *P < 0.05, **P < 0.01

To understand the effects of APOC3 on lipid metabolism in more detail, APOC3-KO and control rabbits were put on an HFD supplemented with 3% cholesterol and 10% soybean oil for 12 weeks at 6 months of age, and their fasting plasma lipid profiles were measured in the 6th and 12th weeks. The plasma of the wildtype (WT) rabbits appeared cloudy due to the high lipid content following high-fat feeding, whereas in the sera of the APOC3 KO rabbits, no obvious chylomicron (CM) aggregation was observed (Fig. 3A). According to the results, the levels of plasma TG, TC, and LDL-C increased significantly, while the levels of plasma HDL-C decreased significantly in all rabbits on an HFD; however, changes in the blood lipid profile of the control group were more obvious than those of the KO group (Table 3). After further analysis, we determined that the levels of TG and LDL-C in the control group plasma increased 13–18 and 17 times, while those in the KO group increased 6–10 and 12 times, respectively. The body weight statistics for the rabbits are shown in Supplementary Fig. 1. Due to the insufficient sample size, it was difficult to judge whether there was a difference in body weight between KO rabbits and WT rabbits after high fat feeding.

**Fig. 4:**
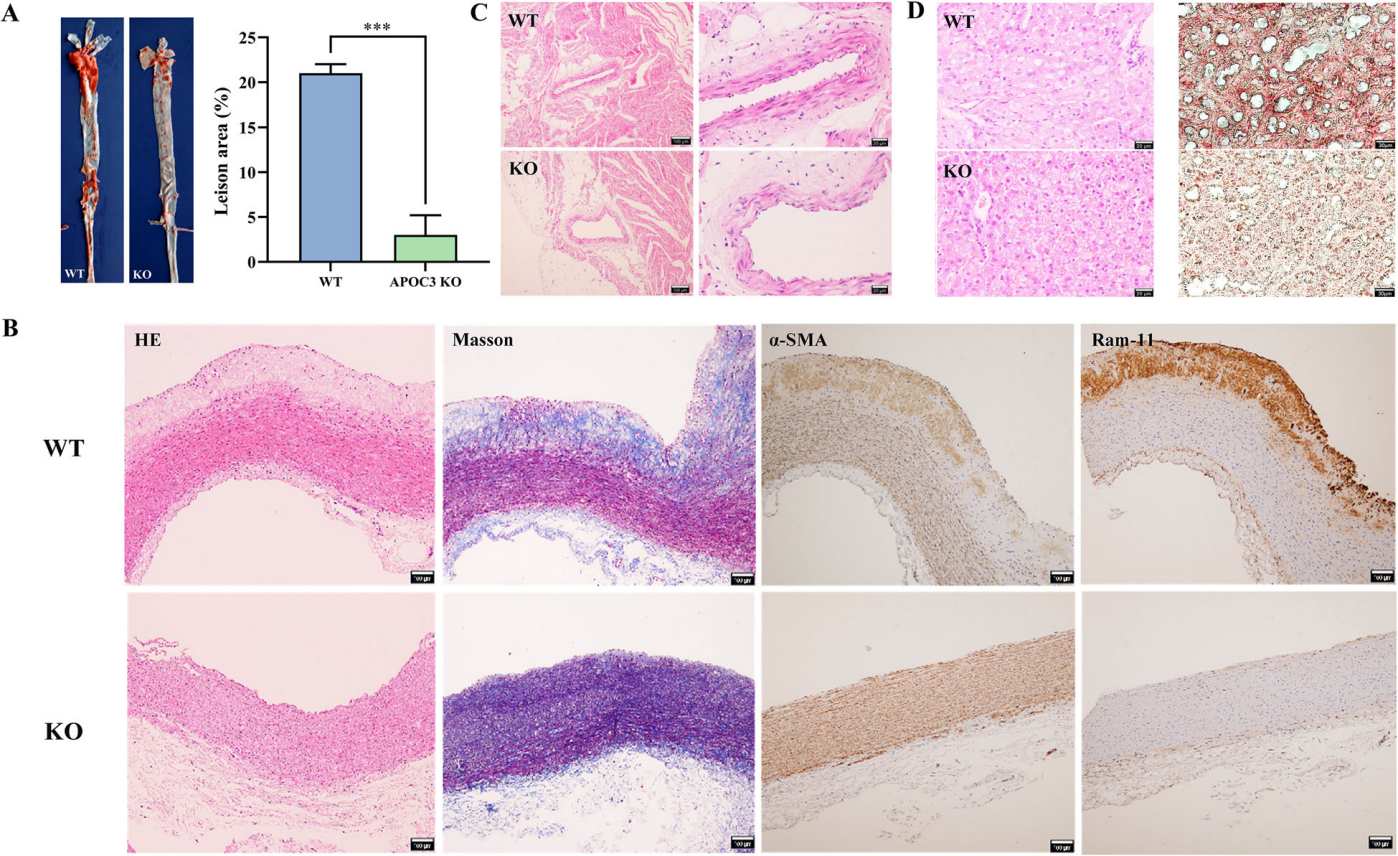
Pathological analysis of atherosclerosis and liver in rabbits. **a** Gross lesions of aortic atherosclerosis of rabbits stained with Sudan IV (visualized as red area) and quantification of the lesion areas. **b** Micrographs of aortic atherosclerosis. Serial paraffin sections were stained with HE and MT, and immunohistochemically labelled with Abs against either RAM-11 for macrophages or α-SMA for SMC (Scale bar = 100 μm). **c** Micrographs of coronary artery. Serial paraffin sections were stained with HE (Scale bar = 100 μm or 20 μm). **d** Micrographs of HE staining of liver (Scale bar = 20 μm) and Oil-red O staining of liver (Scale bar = 30 μm)

We further analyzed the rabbit plasma lipoprotein profiles by agarose gel electrophoresis after 6 weeks of high-fat feeding, and this showed that fewer lipoproteins in the plasma of rabbits AC1-AC3 migrated from their location of origin to the pre-β-area, and more lipoproteins migrated to the α-area than in the control rabbits (Fig. 3A). Consistent with this, western blotting analysis showed significant differences in the plasma lipoprotein profiles of the WT and APOC3-KO rabbits; APOB and APOE were significantly reduced in the APOC3-KO rabbits, while APOA1 was enriched and led to the high HDL levels (Fig. 4B).

According to the results of OFTT test, the plasma TG level in the APOC3-KO rabbits was lower than that of the control group after olive oil gavage (Fig. 3C). Furthermore, after 12 weeks of HFD, plasma LPL activity was significantly increased in KO rabbits compared with control rabbits (Fig. 3D).

### Analysis of atherosclerosis and liver pathological changes

To directly observe the effects of APOC3 on the occurrence and development of atherosclerosis, we assessed the degree of atherosclerotic lesional severity in the coronary artery and aorta of the two groups of rabbits after the HFD regime. As shown in Fig. 4A, the WT rabbits had extensive plaque areas (stained bright red by the Sudan IV dye) in the aortic arch and smaller lesions scattered throughout the aorta. In contrast, only mild lesions were seen in the aorta of the APOC3-KO rabbits. The lesioned areas respectively comprised 21 and 3% of the aortic regions in the WT and APOC3-KO rabbits.

After 12 weeks of providing an HFD to WT rabbits, the HE, MT, anti-RAM-11, and α-SMA staining of the beginning of the aorta showed hyperplastic intima and increased collagen contents accompanied by the accumulation of macrophages and proliferation of smooth muscle cells (Fig. 4B). In comparison, the aortic root lesions of the APOC3-KO rabbits were much milder. However, no significant changes were observed in the coronary arteries of either group (Fig. 4C), likely due to the short duration of the HFD. According to the results of the HE and Oil-red O staining, WT rabbits had significant accumulation of cytoplasmic lipid droplets in their livers, but less damage was observed in APOC3-KO rabbits (Fig. 4D).

### Analysis of plasma inflammatory mediators and full blood count

**Fig. 5:**
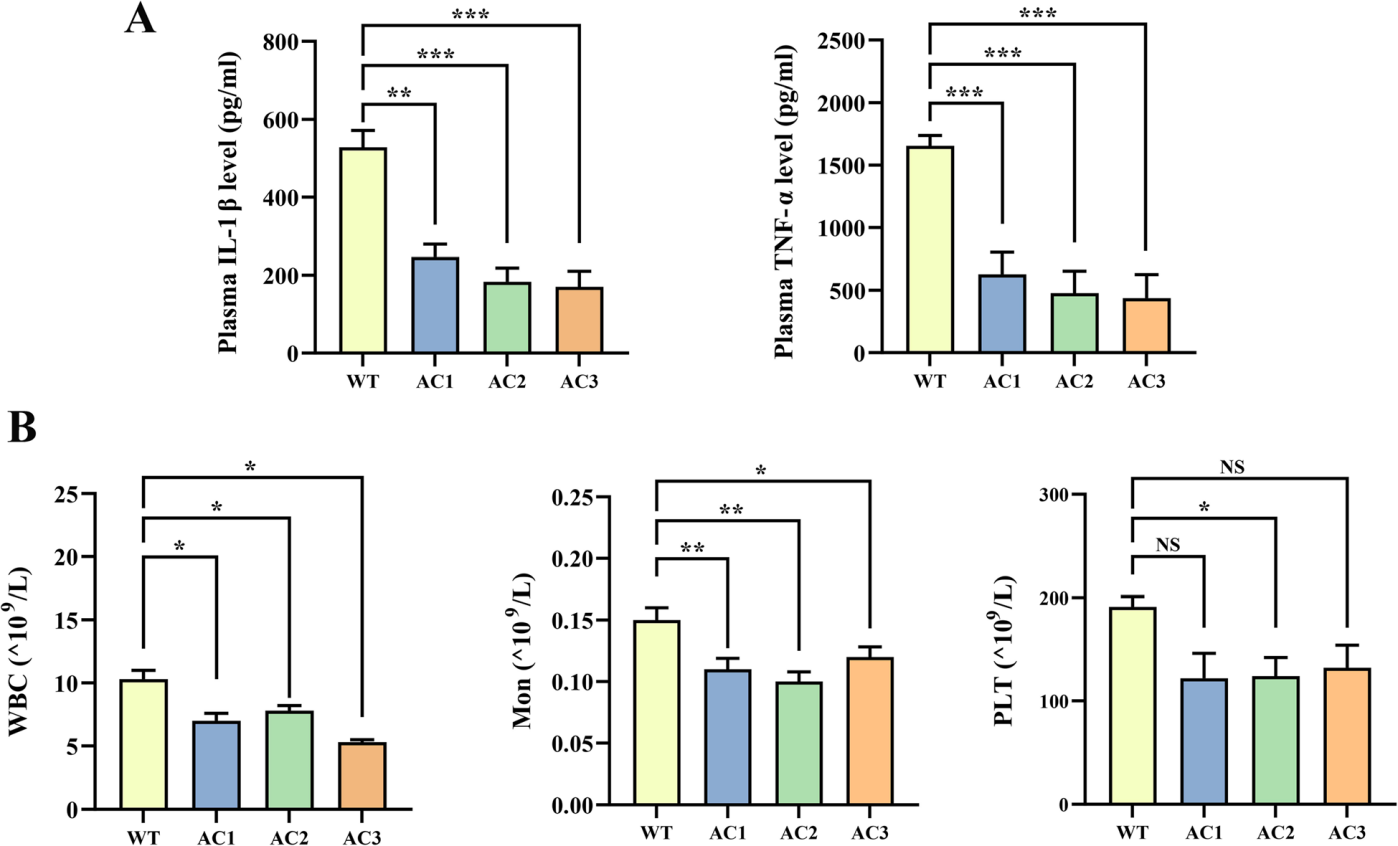
Analysis of plasma inflammatory mediators and full blood count; blood samples were collected three times. Values are expressed as means ± SD., *P < 0.05, **P < 0.01, ***P < 0.001

Atherosclerosis is a chronic inflammatory disease, and the inflammatory process is important in both the initiation and progression of lesion development. Therefore, we also analyzed levels of inflammatory cytokines (IL-1β and TNF-α) and immune cell counts in the rabbits after 12 weeks of HFD. The APOC3-KO rabbits had fewer leukocytes and monocytes in their peripheral blood compared to the WT rabbits in addition to significantly lower serum inflammatory cytokine levels (Fig. 5A and B). Taken together, the evidence indicates APOC3 deficiency not only effectively reduced the number of macrophages in the atherosclerotic plaques but also had an effect on systemic inflammation.

## Discussion

Hypertriglyceridemia is a sign of abnormal lipid metabolism and an independent risk factor in atherosclerotic development [4]. APOC3, which is a key regulator of TG metabolism, is a water-soluble and low-molecular weight lipoprotein ubiquitous in the plasma along with HDLs, VLDLs, CM, and LDLs [22]. Studies show that elevated levels of APOC3 inhibit the activity of LPL and HL, which delays TG-rich lipoprotein clearance and increases TG levels in the plasma, eventually leading to impaired TG metabolism [23]. Although in vivo studies on APOC3 have mainly employed mouse models, rabbit models have several advantages, such as easier maintenance, suitable aortic dimensions, high fecundity, and short gestation periods [24], and they have similar lipid metabolism and cardiovascular pathophysiologies as humans [25]. For instance, the hepatic LDL receptor is normally inactive in rabbits, as in humans, which makes it a highly suitable model for studying the mechanistic basis of AS as well as the effects of lipid-lowering drugs [26]. Furthermore, rabbits have abundant plasma cholesteryl ester transfer protein, which can help when developing strategies to raise plasma HDL-C levels [27]. Finally, both humans and rabbits are more sensitive to an HFD than mice [25, 28].

APOC3 mainly comes from the liver, although the intestine is a secondary source, and it can be secreted into the blood [29]. We designed an sgRNA targeting exon 2 of rabbit APOC3 to finally obtain three KO rabbits by CRISPR/Cas9 gene-editing. None of the three founder rabbits contained APOC3 in their livers and small intestine, and no APOC3 protein was detected in their plasma. Under an NC diet, the plasma TG levels of the KO rabbits decreased, but the plasma TC and LDL-C levels were not significantly different compared with the control group. It is worth noting that after an HFD, in addition to plasma TG levels, plasma TC and LDL-C levels began to differ from the control group, indicating that a lack of APOC3 affects cholesterol transport, and this change is aggravated by a high-fat intake.

The plasma lipoprotein profiles of the WT and APOC3 KO rabbits showed significant differences; while APOB and APOE were significantly reduced in the APOC3 KO rabbits. APOA1 is the main apolipoprotein of plasma HDL [30], so the plasma APOA1 enrichment in KO rabbits leads to the increase of HDL level. This also confirmed that APOC3 may increase plasma TG levels by hindering APOE-mediated uptake of CM and VLDL in the liver by competitively inhibiting APOE binding to its receptor. We detected higher plasma LPL and HL activity in AC1,AC2 and AC3 than in WT rabbits, which is consistent with the results of most studies. However, in a previous study, APOC3 KO rabbits constructed using ZFN technology showed no difference in the activity of these two enzymes compared with the control group [31]. The sample size of our study was limited, and each rabbit had different genotypes, and each KO rabbit was separately divided into a group for statistical analysis. The inconsistent results of the two studies suggest that the correlation between APOC3 and LPL and HL activity needs to be further studied. At present, it is not clear whether APOC3 affects the expression of LPL.

Atherosclerosis is a chronic inflammatory disease, and the inflammatory process is important in both the initiation and progression of lesion development [32–34]. After detection, plasma IL-1β and TNF-α decreased significantly, and the whole-blood monocyte, neutrophil, and platelet counts were significantly lower in KO rabbits than WT rabbits. IL-1β and TNF-α can increase endothelial permeability and lipoprotein permeability [35, 36]. Extensive monocyte recruitment plays an important role in the development of early atherosclerotic lesions [32, 37, 38], while neutrophils aggravate endothelial dysfunction, attract monocytes, enter atherosclerotic lesions, and accelerate the formation of foam cells [39]. Studies have confirmed that platelets contribute to the atherosclerotic process at both the early (endothelial disruption) and final stages (rupture of the vulnerable plaque), participating by releasing chemokines, inflammatory mediators, and microparticles [40, 41]. Therefore, it can be boldly speculated that APOC3 is closely related to the formation and development of atherosclerosis, in which a series of inflammatory responses play an important role.

After 12 weeks of HFD, the aortic tree lesion coverage of the WT rabbits was as high as 21%, while that in KO rabbits was much lower (only 3%). HE, MT, and immunohistochemical signals showed the WT rabbits had obvious atherosclerotic lesions, as well as increased intima thickening and collagen content, accompanied by the accumulation of macrophages and the proliferation of smooth muscle cells, while KO rabbits only had small early atherosclerotic lesions. However, there were no obvious pathological changes in the coronary arteries of the two groups. For the formation of coronary artery plaques in rabbits, higher blood lipids (such as seen in Watanabe heritable hyperlipidemic rabbits or LDLR KO rabbits) or other additional conditional interventions, such as hybridization or surgical ligation, are usually required. Taken together, the results suggest a lack of APOC3 restrains atherogenesis, although the exact mechanistic basis needs to be elucidated.

Statins are often used to treat dyslipidemia, especially in controlling LDL-C elevation; although clinicians generally believe that the benefits of statins are exaggerated, while the potential side effects are underestimated [42]. In addition, other risk factors for atherosclerosis still exist, such as TG and TG-rich lipoproteins [43]. However, the efficacy of existing TG-lowering drugs is still controversial. Therefore, using experimental animal models that are more similar to the characteristics of human lipid metabolism to study the associations between genes and cardiovascular disease may be conducive to the development and application of new drugs.

### Comparisons with other studies and what does the current work add to the existing knowledge

APOC3 KO rabbits have previously been successfully constructed using the ZFN technique with adenine inserted into exon 3 of APOC3 gene [31]. In this study, APOC3-KO rabbits were achieved by anchoring exon 2 using the CRISPR/Cas9 gene editing technique that has becoming commonly used since its recent emergence. The two rabbit models were constructed in different ways. This indicates that the pathways for gene KO are diverse, and the gene target sites are selective.

### Study strengths and limitations

For the first time, APOC3 KO rabbits were produced using the CRISPR/Cas9 system, and the models showed lower lipid levels and fewer atherosclerotic lesions after an HFD. However, only three APOC3 KO primary rabbits were obtained, and their genotypes were not completely consistent, even though, in the end, APOC3 was not detectable in the plasma, liver, or small intestine. The small sample size and different genotypes used were not conducive to the statistical analysis of the experimental data.

## Conclusion

An APOC3-KO rabbit model was constructed using CRISPR/Cas9 gene editing technology. APOC3 deficiency alleviated cholesterol-induced hyperlipidemia and reduced atherosclerotic plaque formation. As the lipid metabolism profile of rabbits is similar to that of humans, this study provides an animal model for basic research on the anti-atherosclerotic effects of APOC3 inhibition therapy, in addition to providing information on the condition for drug development and clinical application.

## Supplementary Information

**Additional file 1: Supplement Fig. 1.** Results of body weight of rabbits before and after HFD.

## Data Availability

The datasets generated and/or analyzed during the current study are available from the corresponding author on request.

## Abbreviations

APOC3: Apolipoprotein C3
TG: Triglycerides
TC: Total cholesterol
LDL-C: Low-density lipoprotein cholesterol
HDL-C: High-density lipoprotein cholesterol
VLDL-C: Very Low-density lipoprotein cholesterol
CM: Chylomicron
APOB: Apolipoprotein B
APOA1: Apolipoprotein A1
APOE: Apolipoprotein E
PCSK9: Proprotein convertase subtilisin/kexin type 9
LPL: Lipoprotein lipase
HL: Hepaticlipase
IL-1β: Interleukin 1 beta
TNF-α: Tumor Necrosis Factor-alpha
OFTT: Oral fat tolerance tests
HFD: High-fat diet
NC: Normal chow
